# Modified Fe_3_O_4_ Magnetic Nanoparticle Delivery of CpG Inhibits Tumor Growth and Spontaneous Pulmonary Metastases to Enhance Immunotherapy

**DOI:** 10.1186/s11671-018-2661-8

**Published:** 2018-08-17

**Authors:** Xueyan Zhang, Fengbo Wu, Ke Men, Rong Huang, Bailin Zhou, Rui Zhang, Rui Zou, Li Yang

**Affiliations:** 1 State Key Laboratory of Biotherapy and Cancer Center/Collaborative Innovation Center for Biotherapy, West China Hospital, Sichuan University, Chengdu, 610041 China; 2 Department of Pharmacy, West China Hospital, Sichuan University and Collaborative Innovation Center for Biotherapy, Chengdu, 610041 China; 3 China West Normal University, No.1 Shi Da Road, Nanchong, 637002 China

**Keywords:** CpG, Magnetic nanoparticles, Intratumoral injection, Immunotherapy

## Abstract

As a novel toll-like receptor 9 (TLR9) agonist, synthetic unmethylated cytosine-phosphate-guanine (CpG) oligodeoxynucleotides can stimulate a Th1 immune response and potentially be used as therapeutic agents or vaccine adjuvants for the treatment of cancer. However, some drawbacks of CpG limit their applications, such as rapid elimination by nuclease-mediated degradation and poor cellular uptake. Therefore, repeat high-dose drug administration is required for treatment. In this work, a CpG delivery system based on 3-aminopropyltriethoxysilane (APTES)-modified Fe_3_O_4_ nanoparticles (FeNPs) was designed and studied for the first time to achieve better bioactivity of CpG. In our results, we designed FeNP-delivered CpG particles (FeNP/CpG) with a small average size of approximately 50 nm by loading CpG into FeNPs. The FeNP/CpG particle delivery system, with enhanced cell uptake of CpG in bone marrow-derived dendritic cells (BMDCs) in vitro and through intratumoral injection, showed significant antitumor ability by stimulating better humoral and cellular immune responses in C26 colon cancer and 4T1 breast cancer xenograft models in vivo over those of free CpG. Moreover, mice treated by FeNP/CpG particles had delayed tumor growth with an inhibitory rate as high as 94.4%. In addition, approximately 50% of the tumors in the C26 model appeared to regress completely. Similarly, there were lower pulmonary metastases and a 69% tumor inhibitory rate in the 4T1 breast cancer tumor model than those in the untreated controls. In addition to their effectiveness, the easy preparation, safety, and high stability of FeNP/CpG particles also make them an attractive antitumor immunotherapy.

## Background

Malignant tumors are one of the major diseases threatening human health and life, and the incidence of tumors is continuously rising [1, 2]. Unfortunately, the outcomes of traditional treatments such as radiotherapy, chemotherapy, and surgery for malignant tumors have not been remarkably effective. In contrast to chemotherapy and radiotherapy, which target rapidly proliferating cells for death, immunotherapies are designed to enhance the host’s own immune defense system to target and eliminate tumor cells.

It is noteworthy that immunotherapeutic CpG have been studied extensively for their efficacy in tumor prevention and regression [3]. Despite encouraging clinical data, the use of free CpG still has several disadvantages. First, CpG are susceptible to nuclease-mediated degradation under biological conditions. Second, they lack specificity to target cells after systemic administration and have poor cellular uptake. Therefore, improvements are likely to be required. Because TLR9s locate on endosomes, we hypothesized that the poor CpG uptake by tumor-associated inflammatory cells causes weak clinical response. Thus, methods that enhance CpG internalization might potentiate its immunostimulatory response.

Except for the drawbacks of free CpG, the route of administration of CpG affects the antitumor ability and toxicity. Free CpG and other stable phosphorothioate oligonucleotides administered by intravenous injection are cleared rapidly and have a broad tissue distribution [4, 5]. Furthermore, systemically administered free CpG can induce a nonspecific immune activation, leading to severe side effects, including immune cell exhaustion, destruction of lymphoid follicles, liver damage, and exacerbation of autoimmune diseases [6–8]. These properties may account for the failure of systemically administered free CpG in human patients [9]. Previous studies have demonstrated that the intratumoral injection of CpG had a great antitumor effect by “focusing” the immune stimulation on tumor sites [10]. How to control the retention time of CpG injected into tumor is a problem.

By using toll-like receptor-9, dendritic cells, macrophages, and NK cells can recognize CpG, which are common in bacterial DNA [11]. CpG induce immune cells to produce chemokines and cytokines, and the upregulated costimulatory cell surface molecules of T cell [12–15]. Thus, CpG have emerged as potent type 1-polarizing adjuvants [16, 17]. Moreover, injecting CpG directly into tumors is a form of immunization that uses the in situ tumor as a source of antigen and introduces CpG as an adjuvant to activate an immune response within the tumor. Therefore, we hypothesized that the intratumor injection of CpG would abolish the immune privilege of tumors via recruiting and activating local dendritic cells, depending on the type 1 antitumor T cell response pathway.

To overcome the aforementioned problems, we developed a modified magnetic particle platform based on Fe_3_O_4_ particles to direct and control the release of CpG through intratumoral injection at tumor sites. Due to some unique characteristics of Fe_3_O_4_ magnetic particles, especially their good histocompatibility [18], superparamagnetism [19, 20], low toxicity [21], and easy preparation and targeted drug delivery with an external magnetic field [22, 23], their movement and concentration can be controlled in the body with an external magnetic field, allowing the Fe_3_O_4_ particles to be used as carriers of gene medicine with enhanced gene transfection efficiency [24]. In addition, coating APTES on Fe_3_O_4_ particles by covalent bonding prevents the formation of aggregates and offers more modification sites (amino groups) [25, 26] for further help in binding CpG. In this study, Fe_3_O_4_ magnetic particles were prepared by a co-precipitation method [27] and modified with APTES. CpG were firmly bonded to the surface of the modified Fe_3_O_4_ particles by electrostatic interaction to form FeNP/CpG particles with a diameter of approximately 50 nm. Further studies showed that FeNP particle delivery systems have an enhanced uptake efficiency of CpG compared to that of free CpG in dendritic cells, and the intratumoral injection of FeNP/CpG particles stimulates an effective antitumor Th1-type immune response not only to restrain the tumor in situ growth but also to inhibit tumor metastasis in C26 colon cancer and 4T1 breast cancer models in vivo. Therefore, nanopreparation therapy may be a potential tumor immunotherapy strategy.

## Materials and Methods

### Materials and Animals

3-Aminopropyltriethoxysilane (APTES) was obtained from Aladdin Company, Inc. Ferric chloride hexahydrate (FeCl_3_∙6H_2_O) and ferrous chloride tetrahydrate (FeCl_2_∙4H_2_O) were purchased from Tianjin Guangfu Fine Chemical Industry Research Institute. The following CpG was synthesized by Invitrogen Co.: 5′-TCGTCGTTTTGTCGTTTTGTCGTT-3′. Fluorescein isothiocyanate (FITC) was purchased from Sigma-Aldrich (St Louis, MO, USA).

A human embryonic kidney cell line (293T), murine breast tumor cell line (4T1), and colon cancer cell line (C26) were obtained from American Type Culture Collection (ATCC), and bone marrow-derived dendritic cells (BMDCs) were obtained from BALB/c mice. Female 6–8-week-old BALB/c mice were purchased from Beijing HuaFuKang Laboratory Animal Co. Ltd. The mice were bred and kept under pathogen-free conditions. All animal protocols were performed according to the Guide for the Care and Use of Laboratory Animals of the National Institutes of Health.

### Preparation of FeNP

The co-precipitation method was used for the synthesis of Fe_3_O_4_ particles [29]. In the experimental procedure, 11.68 g FeCl_3_∙6H_2_O and 4.30 g FeCl_2_∙4H_2_O were added into a three-necked flask containing 200 mL deionized water at 80 °C, followed by the addition of 15 ml 25% NH_3_·H_2_O. During the 1-h reaction process, the mixture was purged with N_2_ and stirred. Thirty milligrams of the prepared Fe_3_O_4_ was dispersed into a mixture of 60 ml deionized water, and absolute ethanol by ultrasonic vibration for 30 min. 0.3 g of prepared Fe_3_O_4_ was dispersed into a mixture of 4 mL deionized water and 600 mL absolute ethanol by ultrasonic vibration for 30 min. Then, 1.2 mL of APTES was added into the mixture under constant mechanical stirring for 7 h. The resulting functionalized APTES-modified Fe_3_O_4_ (FeNP) nanoparticles were magnetically separated from the supernatant by a magnet, then washed with ethanol and dried at 40 °C under vacuum for 24 h. Finally, the precipitate of the FeNP was prepared for further use.

### Characterization of FeNP/CpG Particles

The particle size distribution spectra of Fe_3_O_4_, FeNP, and FeNP/CpG particles were determined using a Zetasizer Nano ZS90 laser particle size analyzer (Malvern Instruments, Malvern, UK) at 25 °C. Each test was conducted three times, and the mean value was taken. A transmission electron microscope (H-6009IV; Hitachi Ltd., Tokyo, Japan) and scanning electron microscope (SEM) were used to observe the morphology of the prepared particles.

An agarose retarding assay was performed to evaluate the CpG binding ability of FeNP particles. Briefly, the functionalized APTES-modified Fe_3_O_4_ particles (FeNP) was mixed with 1 μg CpG at different ratios (FeNP:CpG, *w*/*w*) in distilled water. After co-incubation for 30 min at room temperature, the mixes electrophoresed on a 1% (*w*/*v*) agarose gel at 120 V for 30 min. The gel was then stained with Golden View™ (0.5 mg/mL) and photographed by a UV illuminator (Bio-Rad ChemiDox XRS, USA).

### MTT assay

The cytotoxicity of FeNP particles to C26, 4T1, and 293T cell lines were evaluated with an MTT assay. C26, 4T1, and 293T cells were plated at a density of 5 × 10^3^ cells per well in 100 μL RPMI 1640 medium containing 10% FBS in 96-well plates and grown for 24 h or 72 h. The prepared cells were exposed to a series of FeNP particles at different concentrations: 1.25, 0.625, 0.3, 0.15, 0.07, 0.03, 0.01, and 0 mg/ml in sextuplicate. After cultivating for pointed times, 100 μl complete medium and 10 μl MTT were pipetted into each well and incubated at 37 °C for 4 h. DMSO was added to solute for 30 min, and formazan formed. Then, the absorbance was measured at 570 nm with a Spectramax M5 Microtiter Plate Luminometer (Molecular Devices, USA). The cell viability of the untreated cells was considered 100%.

### In Vitro Transfection

BMDCs were prepared from BALB/c mice. Briefly, bone marrow cells from the femur and tibia were flushed out with free FBS-containing 1640 culture media using a syringe. Cells were centrifuged at 1500 rpm for 3 min, treated with ACK lysis buffer (Lonza Inc.) to remove red blood cells, and resuspended in RPMI-1640 culture medium supplemented with 10% FBS, penicillin (100 U/mL) and streptomycin (100 U/mL) at 37 °C in 5% CO2 with 20 ng/mL granulocyte-macrophage colony-stimulating factor (GM-CSF). The cells were then seeded into six-well plates at a density of 10^6^ cells per well, and the growth medium was changed every 2 days. The dendritic cells were harvested on day 7.

On day 7, a particulate equivalent of 0.2 μg free FITC-conjugated CpG (CpG-FITC) or FeNP delivering CpG-FITC with a mass ratio of 10:1 was added to predesigned wells. One or 3 h post-transfection, the growth media were removed, the cells were stained with DAPI for 10 min, and the cells were washed with physiological saline three times. Pictures of each well were taken with a laser scanning confocal microscope (LSCM), and the transfection efficiency was measured by flow cytometry (NovoCyte Flow Cytometer, ACEA Biosciences, USA).

### In Vivo Tumor Inhibition Assay

One hundred thousand C26 or 4T1 cells were subcutaneously injected on the backs of each BALB/c mouse (6–8 weeks old). The volumes of the tumors were subsequently measured using a digital caliper and calculated by the following formula: tumor volume = 0.5 × length (mm) × [width (mm)]^2^. Once the tumors reached approximately 50 mm^3^ in size, the treatments were applied. The mice (*n* = 10) received either intratumoral injections of CpG/FeNP (at a ratio of 10:1) particulate equivalent to 20 μg CpG or CpG alone every 3 days for four treatments. Mice receiving an equivalent of normal saline (NS) or FeNP particles were regarded as control groups. The tumor size and animal body weight were monitored every 3 days. On day 31, all mice were sacrificed by cervical vertebra dislocation. The tumor tissue and other important organs were fixed with 4% neutral formalin followed by paraffin section for HE staining to evaluate the morphological difference and for immunofluorescence to test tumor microenvironments. In the 4T1 model, we measured tumor weight and counted the number of pulmonary metastasis nodules.

The tumor re-challenge experiment was processed in the C26 model. Briefly, mice cured with the FeNP/CpG treatment were re-challenged with 5 × 10^5^ C26 or 4T1 cells s.c. into two separate sites on the opposite side of the flank without further treatment. Mice were sacrificed when the volume of the 4T1 tumor grew to 200–300 mm^3^ or 20 days.

### Cytotoxic T Lymphocytes Assay

The CTL assay was performed according to published methods. In detail, lymphocytes of the spleen as the effector cells were harvested and depleted of red cells with ammonium chloride and passed through nylon wool at the end of treatment experiments. 4T1 or C26 as target cells were labeled with 300 mci of Na_2_^51^CrO_4_ for 2 h and then washed with PBS and dispensed into 96-well plates. The prepared effector cells were co-incubated with the target cells at different E:T ratios. The supernatants containing radioactivity from the target cells were measured with a scintillation counter. Specific lysis was determined as follows: % specific lysis = 100 [(release in the presence of CTL spontaneous release)/(maximal release spontaneous release)]. In all experiments, the spontaneous release was less than 30% of the maximum release.

### ELISpot Assay

Splenocytes were harvested from control or CpG-treated mice at the end of the experiment, and ELISpot assays were performed using the mouse IFN-γ/IL-4 Dual-Color ELISpot kit according to the manufacturer’s instructions. The harvested splenocytes were co-incubated with their respective treatments for 96 h, the suspension was dropped, and the cells were washed with wash buffer three times before adding the mixed antibody of IFN-γ and IL-4. After being processed by chromogenic agents, the number of spot-forming cells (SFCs) was counted under a microscope.

### ELISA Assay

An ELISA was performed to determine the levels of Ct-specific serum antibody titers according to the manufacturer’s instructions. In brief, micro-plates coated with purified whole Ct protein (BestBio Biotechnology Co. Shanghai, China) were rinsed with PBS solution containing 0.05% Tween 20 (PBST) and then blocked with 100 μl blocking buffer (5% skim milk in PBST) at 37 °C for 1 h. After being rinsed five times with PBST, the plates were incubated with immune sera (diluted at 1:1000 in blocking buffer, 100 μl/well) or vaginal washes (diluted at 1:10 in blocking buffer, 100 μl/well) at 37 °C for 2 h. After being rinsed five times with PBST, the plates were incubated with HRP-conjugated goat anti-mouse IgG antibody (diluted at 1:1000 in blocking buffer, 100 μl/well) or HRP-conjugated goat anti-mouse IgA antibody (diluted at 1:2000 in blocking buffer, 100 μl/well) at 37 °C for 1 h.

### Histological Analysis

The tumor tissue and major organs harvested from in vivo inhibition studies were fixed and embedded in paraffin. After dewaxing and rehydrating, wax-embedded tissue sections were staining with Mayer’s HE. To analyze infiltrating lymphocytes (TILs) within the tumor tissues from each group, sections were subjected to high-pressure antigen repair, stained with anti-mouse FITC-CD4, PE-CD8, and PE-CD49b (as NK maker) (BD, USA) for 1 h at 4 °C, and then stained with DAPI for 10 min prior to PBS washing for imaging. An image from each group was taken through a positive fluorescence microscope (Olympus, Japan).

### Statistical Analysis

Data were expressed as the mean value ± standard deviation. Statistical analysis was performed with a one-way analysis of variance (ANOVA) using Prism 5.0c Software (GraphPad Software, La Jolla, CA, USA) SPSS 17 software. An inter-group comparison was performed using Tukey’s test for the single-factor analysis of variance (ANOVA). *P* values < 0.05 were considered statistically significant.

## Results

### Preparation and Characterization of FeNP/CpG Particles

**Fig. 1:**
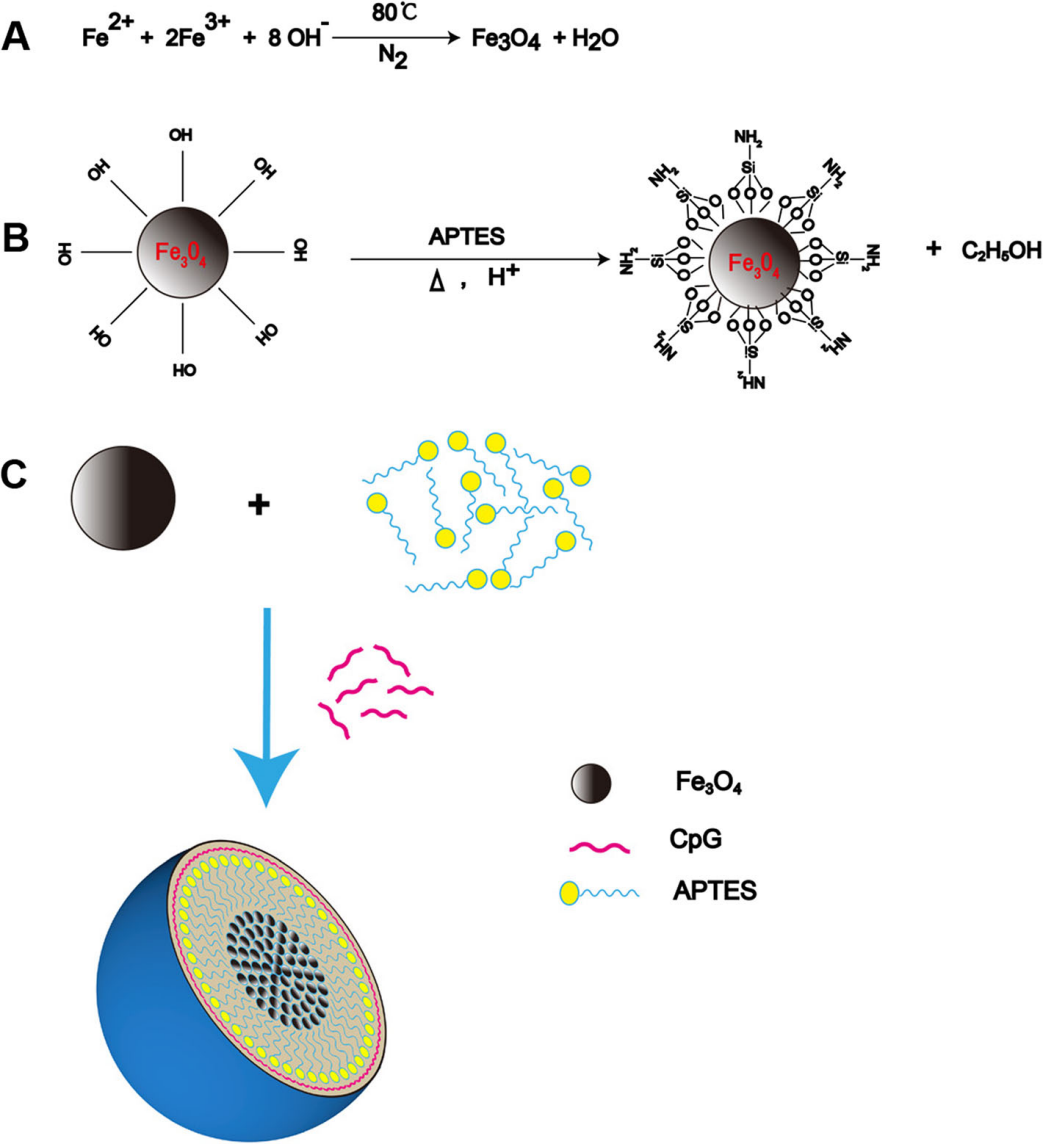
Preparation of FeNP/CpG particles. **a** Equation of synthesis of Fe_3_O_4_ magnetic particles using the co-precipitation method. **b** Hydrolysis and condensation reactions with production of silane polymer and silanization reaction of APTES on the magnetite surface to form FeNP particles. **c** Schematic diagram of the FeNP/CpG particles. CpG are bound to the surface of the FeNPs through electrostatic interactions to form FeNP/CpG particles

**Fig. 2:**
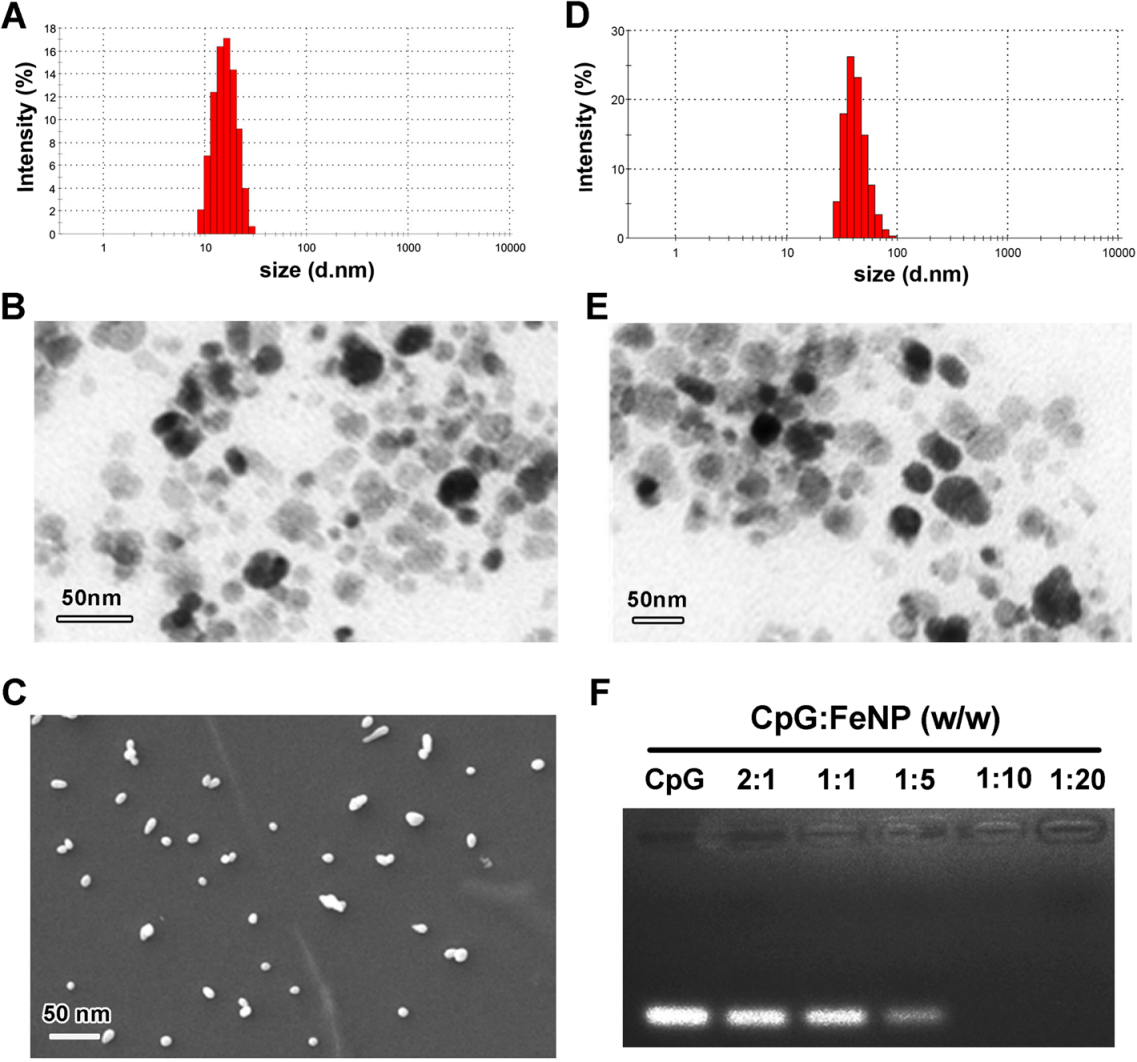
Characterization of FeNP/CpG particles. **a** Size distribution spectrum of Fe_3_O_4_ particles. **b** Transmission electron microscopic image (TEM) of Fe_3_O_4_. **c** Scanning electron microscopy image of Fe_3_O_4_ particles. **d** Size distribution of APTES-coated Fe_3_O_4_ (FeNP) particles. **e** The TEM image of FeNP particles. **f** The CpG binding ability of FeNPs determined by gel retardation assay. All data represent three independent experiments

To develop a magnetic particle for CpG delivery with low cytotoxicity, Fe_3_O_4_ particles were synthesized using the co-precipitation method as shown in Fig. 1a. Grafting the aminopropylsilane groups (–O)3Si–CH2–CH2–CH2–NH2 of APTES on the surface of Fe_3_O_4_ to form FeNP particles (Fig. 1b). Negatively charged CpG (CpG) bind to effective functional groups provided by the APTES to form FeNP/CpG particles through electrostatic interactions as schematically represented in Fig. 1c. Based on the dynamic light scattering measurement, the size of the prepared Fe_3_O_4_ particles was 10.7 ± 4.1 nm (Fig. 2a). As observed through TEM (Fig. 2b) and SEM (Fig. 2c), Fe_3_O_4_ developed in this work displayed a uniform and near-spherical shape. The dynamic diameter of APTES-modified Fe_3_O_4_ particles was 34.5 ± 5.0 nm (Fig. 2d), which was in good agreement with the TEM results (Fig. 2e).

To illustrate the binding ability of FeNP particles to CpG, a gel retarding assay was performed (Fig. 2f). When the molar ratio of FeNP with CpG was 10:1, no bright CpG band was observed, showing the negatively charged CpG can be completely adsorbed by FeNP particles through electrostatic interactions after electrophoresis. Therefore, we chose that prescription ratio for further application in our study. Together, these results demonstrated that CpG can combine successfully on the surface of FeNPs through electrostatic interactions, showing stability and a small dimension.

### Cell Viability and Transfection of FeNP/CpG Particles In Vitro

**Fig. 3:**
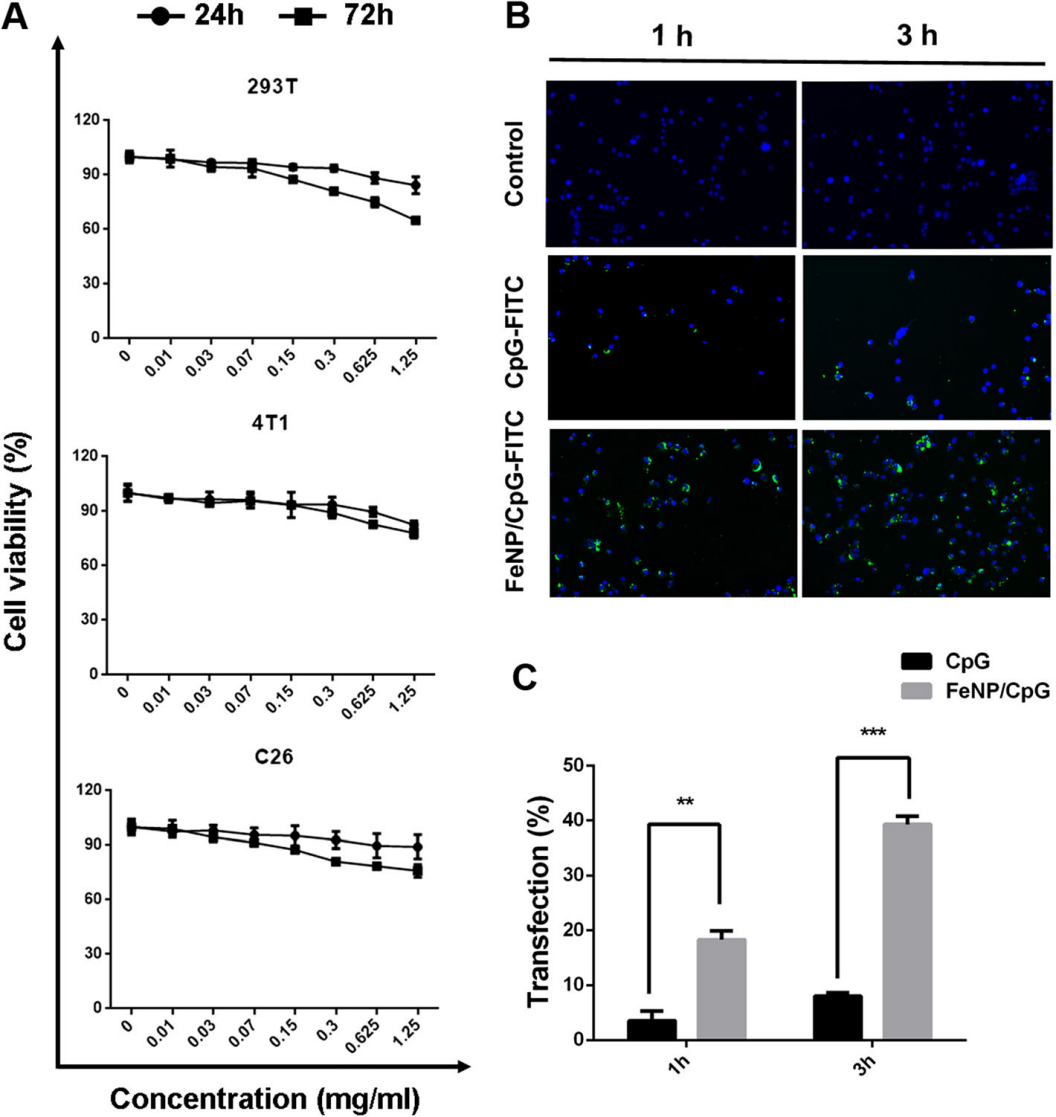
Cytotoxicity and uptake activity of FeNP/CpG particles in vitro. **a** MTT assay. We detected the viability of 293T, 4T1, and C26 cells after treatment with various concentrations of FeNP particles for 24 h or 72 h. There was no significant difference in cell viability at any dose of FeNPs. **b**, **c** The detection of FeNP/CpG uptake in BMDCs. BMDCs prepared after *GM*-*CSF* treatment for 7 days were transfected with CpG-FITC or FeNP-delivered CpG-FITC for 1 or 3 h before being intensively washed, fixed, and labeled with DAPI. Images were captured by laser scanning confocal microscopy (LSCM). Compared with that of free FITC-labeled CpG, the FeNP/CpG with enhanced fluorescence intensity (**b**) and transfection efficiency were statistically significant (**c**). These data were representative of three independent experiments, the mean ± standard error; *P < 0.05, *P < 0.01, and ***P < 0.001 vs the untreated group

To further describe the biophysical characterization in vitro, we detected the cytotoxicity of FeNPs in 293T, 4T1, and C26 cells at the point of 24 h or 72 h (Fig. 3a). There was no obvious dose-dependent relationship between the cell viability and FeNP particles in the 293T, C26, and 4T1 cells. The cell survival rate was more than 80% at 24 h and 60% at 72 h on three kinds of cells, even at a FeNP concentration of 1.25 mg/ml. These results proved the biocompatibility of FeNP particles whether for normal cells (293T) or tumor cells (C26, 4T1).

Dendritic cells as major receptor cells for CpG play an important role in an antitumor immune response mediated by TLR9. CpG as a potent type 1-polarizing adjuvant induces DCs to activate an immune response within the tumor by secreting chemokines and cytokines. Therefore, the efficient delivery of CPG into DC cells is crucial to achieve an antitumor response. In our study, FITC-conjugated CpG were used to investigate the delivery ability of FeNP particles to DCs in vitro. After 1- or 3-h post-transfection, via laser scanning confocal microscopy (LSCM), the cellular fluorescence intensity in the FITC-conjugated CpG-coated FeNP particle (FeNP/CpG-FITC)-treated groups was higher than that for the equivalent amount of free CpG groups (Fig. 3b). The FeNP/CpG-FITC particles were able to transfect up to 18.3% of the DCs after 1 h. Then, 3 h post-transfection, the ratio of the positive cells increased to 39.2% (Fig. 3c). Meanwhile, regarding the free FITC-conjugated CpG-treated cells, minimal fluorescence could be observed even 3-h post-transfection, with less than 10% transfection. Thus, it is suggested that delivering CpG by FeNP particles increased the cellular uptake of CpG.

### FeNPs Deliver CpG to Xenograft and Inhibit Tumor Growth and Spontaneous Pulmonary Metastases In Vivo

**Fig. 4:**
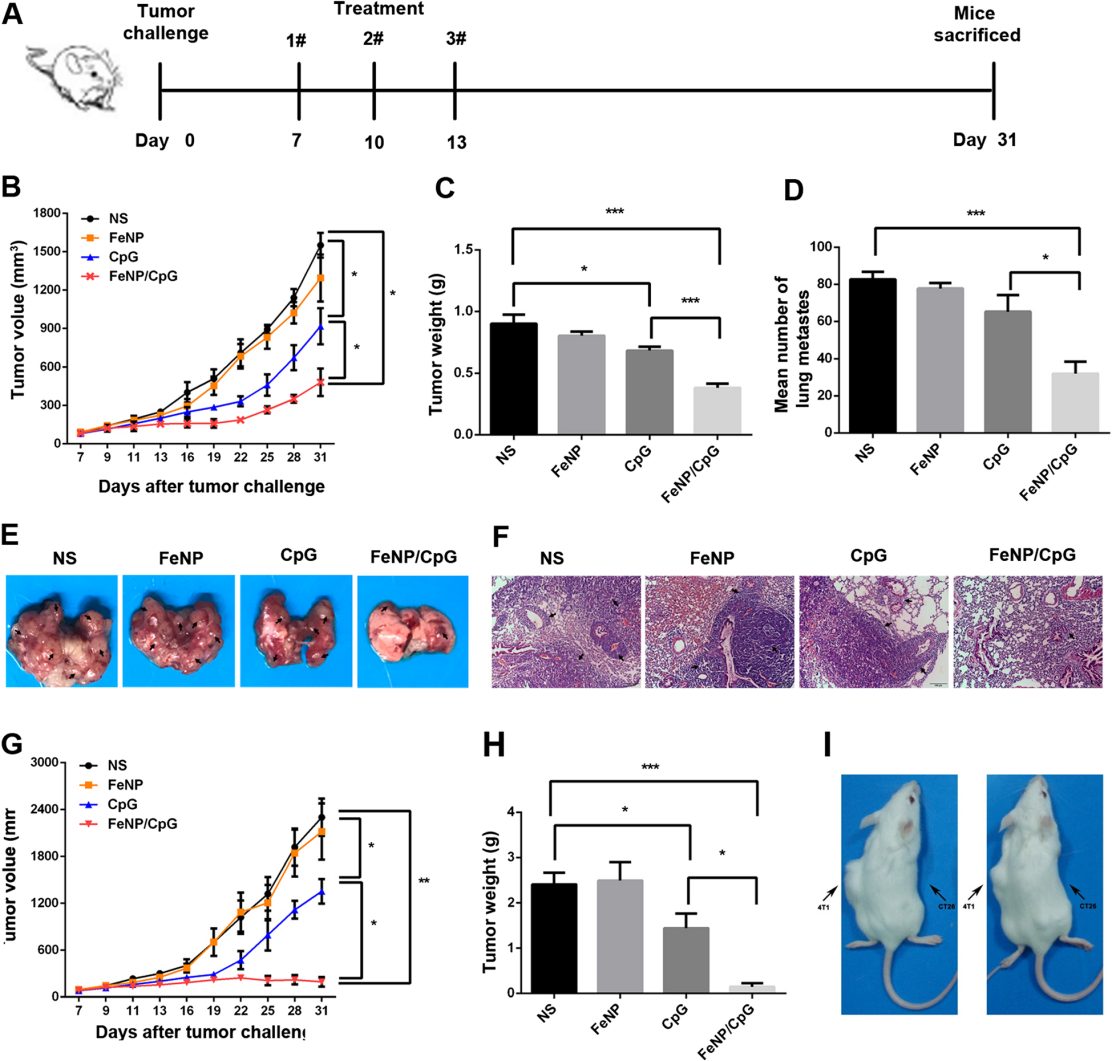
Antitumor ability of FeNP/CpG particles in vivo. **a** Treatment schedule of antitumor by FeNP/CpG particles both in C26 colon cancer and 4T1 breast cancer xenograft models through intratumoral injection in vivo. **b**–**d** FeNP/CpG particle treatment inhibited the growth of 4T1 tumors. **b** Representative tumor growth curves of the 4T1 tumor model. **c** Average tumor weight. **d** Average lung tumor nodule in each group clearly showing lung metastasis after FeNP/CpG intratumoral injection. **e**, **f** Brightfield imaging and H&E staining of the lungs: **e** The number of visible lung metastases, arrows indicate lung metastases. **f** H&E staining of the lung metastasis. Scale bars, 100 μm for H&E staining. **g**, **h** FeNP/CpG particles significantly inhibited the growth of C26 xenograft tumors: **e** tumor growth curves of each group during the experiment and **f** average tumor weight. There were 10 mice in each group. **g** Tumor re-challenge experiment. **i** In C26 model, mice that were cured with FeNP/CpG treatment were re-challenged with 5 × 10^5^ C26 (right flank) or 4T1 cells (left flank) s.c. into two separate sites on the opposite side of the flank without further treatment. Images shown are representative mice at 20 days after tumor re-challenge. All data were representative of three independent experiments, the mean ± SEM; *P < 0.05, *P < 0.01, and ***P < 0.001

The anticancer activity of CpG/FeNP particles was first evaluated in C26 colon cancer and 4T1 breast cancer subcutaneous transplantation tumor models in vivo. When the tumor volume was approximately 50 mm^3^, the mice were randomly divided into four groups to start treatment (*n* = 10). Mice were treated with an intratumoral injection of CpG/FeNP particles three times throughout the entire experiment (Fig. 4a). In our study, intratumoral injection of FeNP/CpG particles resulted in a significant inhibition of xenograft tumor growth compared with that of the control groups, and the inhibitory rate was up to 69% (*P* < 0.05 versus NS) (Fig. 4b). Compared with the normal saline (NS) treatment group (0.90 ± 0.08 g) and the FeNP group (0.81 ± 0.03 g), the FeNP/CpG particle-treated groups experienced a statistically significant reduction in tumor weight (0.38 ± 0.03 g, *P* < 0.001). Comparatively, the free CpG group showed minimal anticancer ability (0.68 ± 0.03 g) (Fig. 4c). In addition, mice in the PBS control group developed extensive pulmonary metastases, in comparison, those treated with the FeNP/CpG particles showed a 64.1% decrease in the number of tumor nodules in the lung (Fig. 4d, e), demonstrating the power of the FeNP/CpG particles in treating metastatic tumors. As shown in Fig. 4f, H&E staining of the lung showed reduced lung burden with clearly pulmonary metastases after FeNP/CpG treatment than other groups. These results indicate that FeNP delivery CpG into 4T1 cells not only significantly inhibited 4T1 tumor growth (*P* < 0.001 versus NS) but also suppressed the metastasis of breast cancer to the lungs.

Similarly, a significant enhanced antitumor effect with FeNP/CpG treatment groups was observed in the subcutaneous model of C26-incubated mice. As shown in Fig. 4g, the tumor grew rapidly in the control mice, vehicle group, and CpG group, with mean tumor volumes of approximately 2300 ± 239.4 mm^3^, 2116.7 ± 360.9 mm^3^, and 1353.3 ± 158.9 mm^3^, respectively, by day 31. In contrast, in the FeNP/CpG group, the tumor growth was extremely inhibited, with a mean tumor volume of approximately 153.7 ± 62.7 mm^3^ (*P* < 0.01 versus NS) and an inhibitory rate of 94.4%. The tumors seemed to grow slowly after the initial treatment, and a part of the tumors begin to disappear gradually after the end of the three treatments. Even more importantly, in the FeNP/CpG particle treatment groups, the tumors in nearly half of the mice completely had disappeared by the end of the experiment. As shown in Fig. 4h, the FeNP/CpG treatment group had a much lower average tumor weight than that of the other groups (*P* < 0.001): FeNP/CpG treatment group (0.14 ± 0.08 g), NS group (2.41 ± 0.26 g), FeNP group (2.50 ± 0.4 g), and CpG group (1.44 ± 0.32 g). To determine whether FeNP/CpG particles were sufficient to mediate the specific antitumor response, a tumor re-challenge experiment was processed in the C26 model (Fig. 4i). The mice whose tumors were completely regressive after the FeNP/CpG particle treatment in the C26 colon cancer model were inoculated with 4T1 and C26 tumors in different positions on the BALB/c mice subcutaneously, and no further treatment was given. All the 4T1 tumors grew rapidly, and their volumes surpassed 1000 mm3 by day 20 post-challenge. In contrast, there were solid tumors that were invisible to the naked eye in the C26 model, suggesting the FeNP/CpG particles groups stimulated a specific antitumor response.These results suggested that the intratumoral injection of FeNP/CpG particles efficiently inhibited the growth of a subcutaneous xenograft.

### FeNP/CpG Particles Stimulate Enhanced Systematic Antitumor Immune Response

**Fig. 5:**
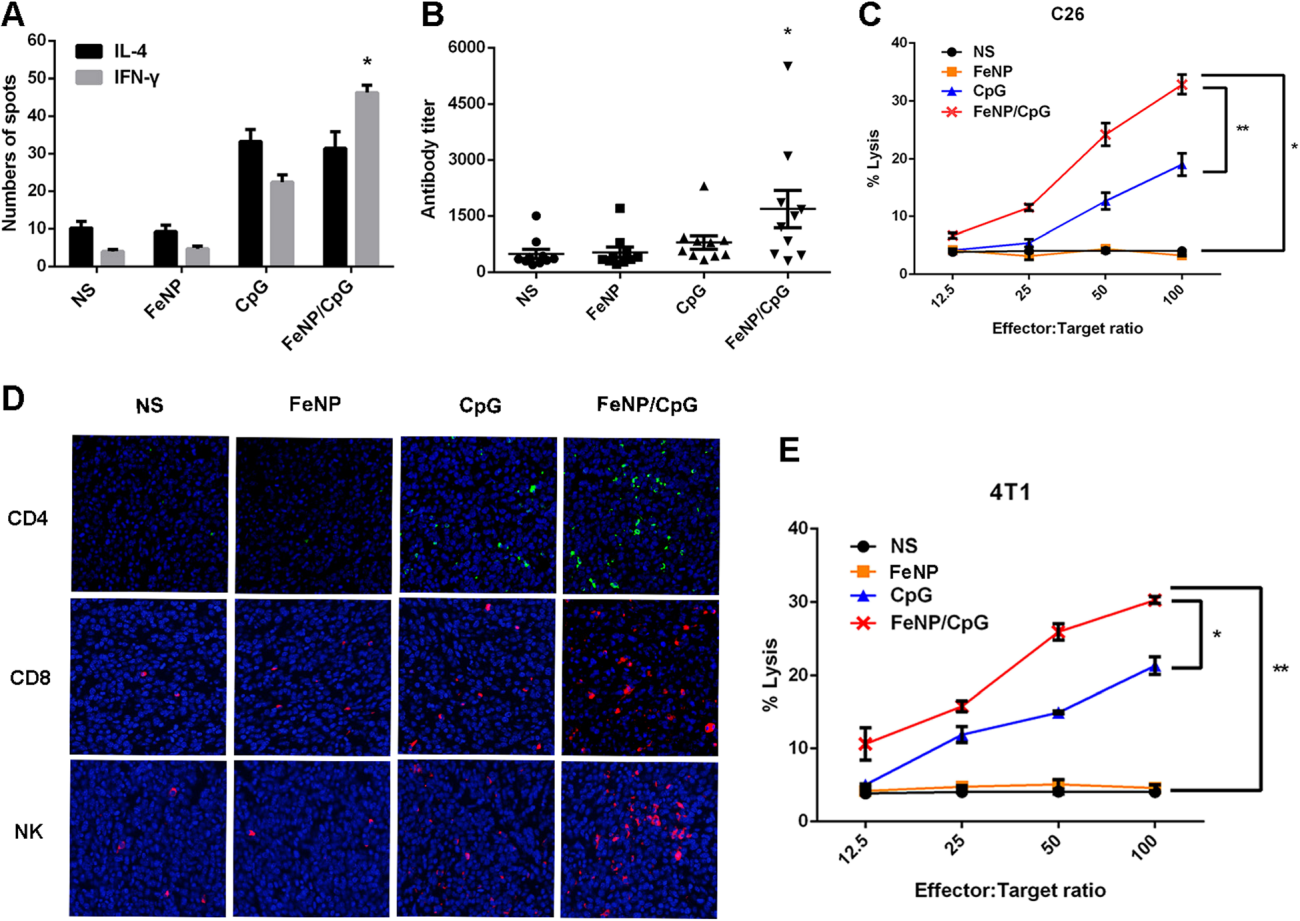
The mechanism of antitumor immune response. **a** ELISpot assay. Splenocytes were harvested from NS or treated mice after the final treatment, and ELISpot assays were performed using the mice IFN-γ/IL-4 Dual-Color ELISpot kit in the C26 xenograft model. **b** In the C26 model, serum antibody titers in tumor-bearing mice treated by FeNP/CpG particles were tested using an ELISA assay. Each symbol represents an individual mouse, and horizontal lines indicate the median as determined by Mann–Whitney *U* tests. **c** CTL assays. The cytotoxicity of splenocytes against C26 cells were examined in a 4-h 51Cr-release assay. **d** Representative immunofluorescence images showing infiltrating immune cells in tumor tissues in each group. The FeNP/CpG particles treatment groups with enhanced CD4^+^T, CD8^+^T and NK (CD57^+^) cell infiltration in tumors compared to that of other groups. **e** The cytotoxicity of splenocytes against 4T1 cells were examined in a 4-h 51Cr-release assay

The in vivo antitumor mechanisms of FeNP/CpG particles in the above two models were further studied. To study the type of immune response, we applied the ELISpot assay to analyze the IFN-γ/IL-4 levels of the mouse spleen. If the IFN-γ level in spleen lymphocytes (showing erythema on the ELISpot board) is significantly more than the IL-4 level (showing blue spots in ELISpot board), the type of immune response stimulated was Th1, namely, to activate CD8^+^T lymphocytes and mainly the body’s CTL response. Otherwise, the type of immune response was Th2, namely, to mainly activate CD4^+^T lymphocytes, thus stimulating B lymphocytes and producing antigen-specific antibodies. A dual-color ELISpot assay was conducted to measure the expression of IFN-γ and IL-4 in different treatments (Fig. 5a). Compared with that in the other three groups, the number of spot-forming cells (SFCs) of IL-4 and IFN-γ secreted from mice spleen lymphocytes had an ascending trend in the FeNP/CpG particle treatment group, and the number of SFCs secreting IFN-γ was 1.5-fold greater than that for IL-4 (*P* < 0.05), which implied an enhanced antitumor cellular immune response after FeNP/CpG particle intratumoral injection. In addition, the serum from the NS, FeNP, CpG, and FeNP/CpG-treated mice was analyzed using an ELISA assay for the presence of total tumor-specific IgG after three immunizations. In the C26 tumor model, although FeNP/CpG-immunized mice developed significantly higher titers of tumor-specific IgG than those in other groups (Fig. 5b), there was no statistical difference between FeNP/CpG-immunized mice and CpG-immunized mice.

To assess the cell-mediated immune response stimulated by the FeNP/CpG, CTL activity was assessed using the 51Cr release assay on C26 and 4T1 cells ex vivo (Fig. 5c, e). When the killing activity of CTL was at a 100:1 ratio of effector cells to target cells, in the C26 cells, the effectors in the FeNP/CpG groups showed 8.2-fold more tumor-killing activity than that in the NS group and 9.7-fold and 1.7-fold more than that in the FeNP and CpG groups (*P* < 0.05 versus NS) (Fig. 5c). Similarly, this observation was coincident with the result in the 4T1 cells; the FeNP/CpG group had 7.4-fold more tumor-killing activity than that of the NS group and 6.6-fold and 1.4-fold more than that of the FeNP and CpG groups (*P* < 0.01 versus NS). These results illustrated that compared to that of free CpG, the use of APTES-coated Fe_3_O_4_ to deliver CpG can stimulate a more intense humoral immune response and result in a better antitumor efficiency in vivo.

### The FeNP/CpG Increases Infiltrating Lymphocytes in Tumors and Alters Tumor Microenvironments

**Fig. 6:**
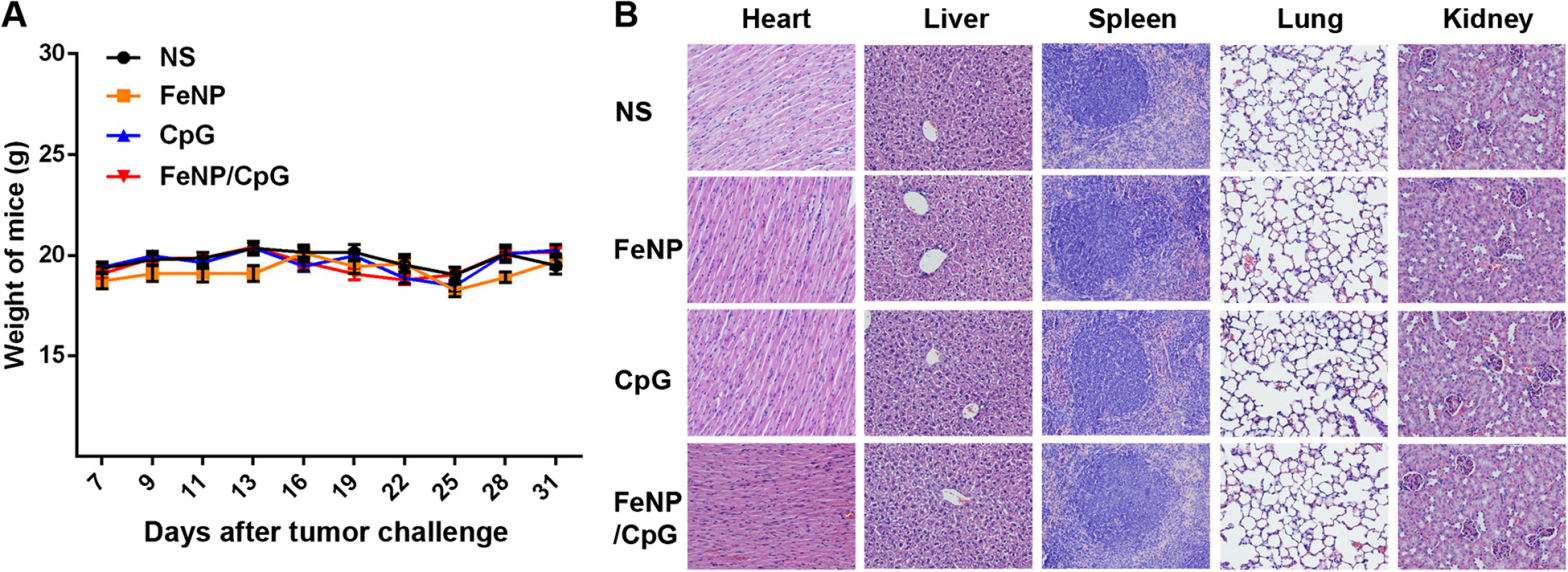
Safety and toxicity evaluation of FeNP/CpG formulation. (**a**) Body weight changes in NS, FeNP, CpG, and FeNP/CpG groups. **b** The section of the heart, liver, spleen, lung, and kidney in each group was collected and stained with H&E staining. No significantly pathological changes were observed in any group (magnification, × 200)

Tumor-infiltrating lymphocytes (TILs), a primary immune component infiltrating solid tumors, are considered the manifestation of the host antitumor reaction. Immunofluorescence analyses were performed to study TILs in tumors. The intensity of infiltration by CD4^+^T cells, CD8^+^T cells and CD49B^+^ NK cells was studied (Fig. 5d). There was a significant increase in the intensity of infiltration of NK cells, CD4^+^T cells, and CD8^+^T cells in tumors from the FeNP/CpG groups compared with that in the FeNP, CpG, and NS groups (*P* < 0.005), which suggests that FeNP/CpG not only stimulates a higher immune response but also alters the tumor microenvironment by activating more immune cells into the tumor tissues. During the entire experiment, the mouse body weight and state were observed, and there was no piloerection, poor appetite, weight loss, or abnormal behavior in the C26 xenograft model (Fig. 6a). As shown in Fig. 6b, no significant pathological changes in heart, liver, spleen, lung, or kidney were observed through HE analysis. No histopathological changes and changes of body weight were observed in the 4T1 subcutaneous xenograft model (data not shown). Overall, our data suggested that the developed FeNP particles for CpG delivery were capable of treating cancer and inhibiting tumor metastasis with high safety.

## Discussion

Synthetic CpG, identified as an effective drug in immunotherapy by simulating the immune activation of natural bacterial DNA [28–30], have been characterized in a variety of tumor models. CpG intratumoral injection is one of treatments in clinical trials on CpG. Molenkamp and his colleagues showed that the intratumoral injection of CpG alone or combined with radiotherapy could obviously increase the tumor-specific CD8 ^+^T cell immune response in lymphoma or melanoma patients, causing systemic tumor regression [31]. So far, CpG combined with other treatments showed both a great antitumor effect and good security, which suggests that CpG are quite effective in tumor immunotherapy. However, the greater challenge of CpG truly coming into clinical application is if they can efficiently be delivered into the cells to combine with receptors such as TLR9 to stimulate an immune response. Nanotechnology can address such concerns by enhancing the delivery of CpG to antigen-presenting cells, and a number of nanocarriers have been explored for this purpose [32–34]. The nanoparticle formulations explored include gelatin particles, liposomes [35, 36], and DNA origami structures, but these were only explored in the context of combination treatments.

In this study, we first developed a non-viral delivery system, FeNPs, which were easily formed via APTES-modified Fe_3_O_4_, to deliver CpG into BMDCs. The CpG surrounded the surface of the modified magnetic Fe_3_O_4_ particles via electrostatic interactions to form FeNP/CpG particles with a small size distribution. The FeNP/CpG particles not only showed enhanced transfection efficiency compared to that of free CpG but also had a great antitumor effect in subcutaneous tumor models of C26 colon cancer, with 94.5% tumor inhibition, and 4T1 breast cancer, with a 64.3% inhibitory rate, though intratumoral injection in vivo. Moreover, FeNP/CpG treatments significantly stimulate an effective antitumor immune response and alter the tumor microenvironment by recruiting a number of immune cells to tumor tissues.

We further discuss possible reasons to account for the enhanced transfection and therapeutic efficiency of FeNP/CpG particles over that of free CpG. First, the developed FeNPs take advantage of characteristics such as their good histocompatibility, superparamagnetism, and a strong positive charge (for the Fe_3_O_4_ particles) to assist in delivering the CpG into the intracellular space, resulting in drug-carrier particles focusing on the tumor site to increase the drug concentrations in the area, reduce the drug loss, and improve drug utilization. Simultaneously, APTES was used to modify Fe_3_O_4_, providing more CpG binding sites to firmly bind the CpG onto the carrier. FeNPs exerted higher cell viability on 293T, C26, and 4T1 cells in vitro, and there was no remarkable pathological change after FeNP/CpG treatment by intratumoral injection that could avoid the side effects due to CpG entering into circulation by systemic administration. Second, the FeNP load CpG into DCs with a higher transfection efficiency than that of free CpG, increasing opportunities for integration with intracellular TLR9, which is widely expressed in macrophages, NK cells, DCs, and so on. The combination of CpG and TLR9 induces the secretion of local cytokines and chemokines, recruiting and activating immune cells of the innate immune response to stimulate the antitumor immune response; additionally, NK cells and CTLs could kill tumor cells directly or use IFN-*γ* or granzyme B to kill tumor cells [37, 38]. We suspected that FeNP/CpG may be taken up by the recruited immune cells to trigger the secondary cascade amplification of the immune response. Third, some studies have indicated that CpG can react with tumor cells expressing TLR9, aiming to induce tumor cell autophagy [39]. Autophagy may enhance the sensitivity of tumor cells to the immune response, and the ATP dependent on autophagy could be released extracellularly and used to recruit DCs into the tumor tissue, activating the tumor-specific T cell immune response. This therapy is a form of immunization that uses the in situ tumor as a source of antigen and introduces CpG as an adjuvant to activate an immune response within the tumor. In our study, although the FeNP/CpG was injected without any specific tumor antigen, a cell-specific and systematic immune response could be elicited, and the contralateral tumors appear regressive, as shown in the tumor re-challenge experiment and the two-tumor site model. In addition, being coated with the lysate of tumor cells in a 96-well plate, the ELISA assay showed after FeNP/CpG intratumoral injection in mice that the antitumor antibody titer in the serum appeared to significantly increase. The ELISpot assay suggested that FeNP/CpG treatment, to a great degree, increased the spleen lymphocyte secretion of IFN-*γ* and IL-4, especially that of IFN-*γ*, which indicated that the humoral immunity responses and cellular immunity responses were both enhanced by treatment with FeNP/CpG in mice. In addition, immunohistochemistry analyses were performed to study TILs in the tumor microenvironment. Compared to that in the other groups, in the FeNP/CpG group, the number of CD4^+^T, CD8^+^T, and NK cells in the tumors showed a significant increase. Taken together, our study suggested that FeNP/CpG particle intratumoral injection may be a potential tumor immunotherapy strategy.

In recent years, treatment strategies based on nucleic acids have become one of the important areas of tumor treatment. The negative charge of the lipid bilayer of cell membranes makes it difficult for nucleic acid drugs with the same charge to pass through the cell membrane into the cell. Thus, APTES-modified Fe_3_O_4_ as a safe and efficient delivery system is expected to be used for other nucleic acid drugs to contribute to the carrier solution.

## Conclusion

In summary, magnetic APTES-modified Fe_3_O_4_ particles (FeNP) were used to deliver CpG adjuvants via intratumoral injection to treat C26 colon carcinoma and 4T1 breast cancer, with enhanced antitumor ability over that of free CpG. The prepared FeNPs showed high stability, low toxicity, and high transfection ability for CpG in vitro and in vivo. Our results demonstrated the potential capacity of FeNP particles in non-viral nucleic acid drug delivery and offered an alternative strategy for CpG immunotherapy.

## Abbreviations

APTES: 3-Aminopropyltriethoxysilane
BMDC: Bone marrow-derived dendritic cell
CpG: Cytosine-phosphate-guanine
CpG-FITC: FITC-conjugated CpG
FeNP: 3-Aminopropyltriethoxysilane-modified Fe_3_O_4_ nanoparticle
FeNP/CpG: FeNP-delivered CpG particles
FeNP/CpG-FITC: FeNP-delivered CpG-FITC particles
*GM*-*CSF*: Granulocyte-macrophage colony-stimulating factor
